# Tit-For-Tat Strategy for Increasing Medical Student Evaluation Response Rates

**DOI:** 10.5811/westjem.2017.9.35320

**Published:** 2017-12-07

**Authors:** Matthew G. Malone, Michelle M. Carney, Joseph B. House, James A. Cranford, Sally A. Santen

**Affiliations:** * The Ohio State University College of Medicine, Department of Emergency Medicine, Columbus, Ohio; † University of Michigan Medical School, Department of Emergency Medicine, Ann Arbor, Michigan; ‡ University of Michigan Medical School, Department of Pediatrics, Ann Arbor, Michigan; § University of Michigan Medical School, Department of Psychiatry, Ann Arbor, Michigan

## Abstract

**Introducation:**

It is essential for faculty to receive feedback on their teaching for the purpose of improvement as well as promotion. It can be challenging to motivate students to provide feedback to preceptors and fill out evaluation forms when not a clerkship requirement. Furthermore, there is concern that making the evaluations a requirement can compromise the quality of the feedback. The objective of this study was to identify an increase in the number of faculty and resident evaluations completed by students rotating through their Emergency Medicine clerkship following the implementation of a tit-for-tat incentive strategy.

**Method:**

Prior to the implementation of Tit-for-Tat, students rotating through their emergency medicine clerkship were asked to fill out evaluations of residents and faculty members with whom they worked. These were encouraged but voluntary. Beginning in the 2014–2015 academic year, a tit-for-tat strategy was employed whereby students had to complete a resident or faculty evaluation in order to view the student assessment completed by that resident or faculty preceptor.

**Results:**

Students submitted 1101 evaluations in the control, with a mean of 3.60 evaluations completed per student and 3.77 evaluations received per preceptor. Following the implementation of tit-for-tat, students submitted 2736 evaluations, with a mean of 8.19 evaluations completed per student and 7.52 evaluations received per preceptor. Both the increase in evaluations completed per student and evaluations received per preceptor were statistically significant with p-value <0.001.

**Conclusion:**

The tit-for-tat strategy significantly increased the number of evaluations submitted by students rotating through their emergency medicine clerkship. This has served as an effective tool to increase the overall number of evaluations completed, the number of evaluations each instructor received on average and the proportion of students that completed evaluations. Further work could be done to attempt to better assess the quality of the feedback from these evaluations.

## INTRODUCTION

Student evaluations are paramount to faculty both administratively and academically. Evaluations have been used as data to inform the decision for promotion and tenure in higher education for years.^1^ By comparing data of faculty obtained through trainee evaluations, individual educator performance can be measured. Equally as important is the ability for faculty to grow as educators by internalizing feedback from evaluations; celebrating accomplishments and providing a substrate for areas in which growth is necessary.^2^ Using student evaluations in this way employs the social constructivist model; faculty use feedback from students for professional development and reflective improvement. Knowledge and behavior are built through interaction and feedback from others.^3^

Online evaluations have become an increasingly popular method of obtaining evaluation data.^4^ Previous work has identified significant advantages to the online evaluation model, which include potentially significant cost savings, improved turnaround time, greater elaboration afforded by typed responses and convenience for students to respond without using valuable class time.^5^,^6^ In addition, online evaluations are often the preferred method by students.^7^

However, online methods of evaluation are not without disadvantage. It is well established that converting from paper evaluations to an online evaluation system results in lower response rates, which in turn can lead to increased bias and less valuable data.^8^,^9^

Online evaluations may be more convenient, but literature is lacking in how to motivate students and trainees to complete online evaluations. To bridge this gap, the authors tried to make the completion of evaluations a tit-for-tat situation. If the students wanted to see their evaluations during the rotation and prior to receiving their grade, they must complete an evaluation for their supervisor. In this way, the motivation comes from an internal need for feedback. The objective of this study was to identify an increase in the number of faculty and resident evaluations completed by students rotating through their Emergency Medicine clerkship following the implementation of a tit-for-tat incentive strategy. The authors hypothesize that a tit-for-tat strategy whereby students had to complete a resident or faculty evaluation in order to view their student assessment completed by their resident or faculty preceptor would increase the total number of preceptor evaluations.

## METHODS

This study was a retrospective cohort study of medical student evaluations of faculty and resident preceptors before and after the implementation of a tit-for-tat method to increase the number of total evaluations completed. The (blinded) Institutional Review Board approved the study.

As part of the required fourth-year Emergency Medicine clerkship at (blinded), students were asked to fill out evaluations of residents and faculty members with whom they worked. These evaluations were encouraged but voluntary. Prior to the 2014–2015 academic year, students would receive online assessments from faculty and resident preceptors. Similarly, faculty and residents would receive online evaluations from medical students with whom they worked. These evaluations of faculty and residents were blinded and aggregated so that the preceptor could not identify the medicals student.

Beginning in the 2014–2015 academic year, a tit-for-tat strategy was employed whereby each student would receive an online push notification that an assessment from a resident or attending physician had been completed and in order to view this assessment, the medical student had to complete an evaluation of the resident or attending physician in order to see the assessment of their performance. The assessment of the medical student by resident or faculty thus became un-blinded to ensure that the students knew which evaluations to complete in order to view their own assessment from the preceptor. This method was thought to not introduce bias, as the preceptor’s assessment of the student could not be viewed until the student submitted the evaluation of that preceptor. Further, the evaluations completed by medical students remained blinded, such that the preceptor could not identify the medicals student evaluator. The authors could not identify any other changes in the evaluation process that would confound the results. This strategy was employed to increase the total number of resident and faculty evaluations completed by medical students.

**Population Health Research Capsule**

What do we already know about this issue?

Methods to increase online survey and evaluation response rates have been applied in the commercial and undergraduate education literatu e, but rarely studied with medical students.

What was the research question?

Does an incentive strategy increase the response rate of preceptor evaluations completed by medical students?

What was the major finding of the study?

There was an increase in evaluations completed by medical students following the implementation of an incentive strategy.

How does this improve population health?

More feedback to educators hopefully leads to better educators, educational materials and methods. Better education leads to better health care providers and healthier populations.

In both the control cohort and the tit-for-tat cohort, all evaluations were submitted by three weeks after the completion of the rotation. Once the grade was assigned, 3–4 weeks after the clerkship, students could view all of their assessments of performance, as it was felt to be unfair to completely withhold feedback information.

The total number of student evaluations of both resident and faculty was recorded from 2014–16 following the implementation of tit-for-tat, as well as from 2012–2014, which was used as a control. A Chi-squared analysis was performed to demonstrate a statistically significant increase in the number and proportion of medical students who chose to fill out evaluations. The mean number of evaluations completed per student per academic year and the mean number of evaluations received per preceptor per academic year were also calculated and compared.

## RESULTS

In the control cohort, 201 of the 306 rotating medical students completed a total of 1101 of evaluations of faculty and resident preceptors. In the tit-for-tat cohort, 307 of the 334 rotating medical students completed a total of 2736 of evaluations of faculty and residents (Table 1). In the control cohort, 64.0% of rotating students completed at least one evaluation. In the tit-for-tat cohort, 91.3% of rotating students completed at least one evaluation. A Chi-squared analysis was performed and there was a statistically significant increase in student participation in completing evaluations following the implementation of tit for tat, x^2^ (1) = 69.8, p < .05.

The mean number of evaluations completed per student was calculated from 2012–2016 to control for the variation in number of medical student rotators between academic years. An independent samples t-tests was performed to demonstrate a statistically significant increase from the control cohort to the tit-for-tat cohort. The mean number of evaluations completed per student was 3.60 (SD = 3.959) in the control cohort. The mean increased to 8.19 (SD = 3.791) evaluations completed per student in the tit-for-tat cohort, which was statistically significant with p-value <0.001. This statistically significant increase in evaluations completed per student is maintained for both faculty and residents when calculated independently (Table 2).

In addition, the mean number of evaluations received per preceptor was also calculated to control for variation in the number of resident and faculty between academic years. The mean number of evaluations received per preceptor increased from 3.77 (SD = 2.743) in the control cohort to 7.52 (SD = 5.599) evaluations per preceptor in tit-for-tat cohort, which was statistically significant with p-value <0.001. Again, this increase is maintained for both faculty and residents when calculated independently (Table 3).

## DISCUSSION

The term “tit-for-tat” is an English saying dating to 1556 meaning an equivalent to an action given in return.^10^ While this often carries a negative connotation, such as a blow for blow retaliation, tit-for-tat has also been used to describe positive symbiotic relationships, such as reciprocal altruism.^11^ In the student-preceptor relationship, the responsibility of the preceptor is to provide feedback; likewise it is the student’s role to reciprocate. Faculty evaluations are used to recognize and reward excellence as well as to identify outliers in performance and provide feedback to facilitate reflective improvement.

It is well established in the current literature that converting from paper evaluations to an online evaluation system results in lower response rates.^8^ Further, it has been suggested that response rates are themselves a critical indicator of both student and faculty engagement in the course.^4^ With lower response rates, the potential for error in any survey increases and in turn, the reliability of the data tends to weaken as response rates decline.^9^ It has also been shown that respondents and non-respondents to evaluations differ. For instance, students are more likely to complete evaluations in courses where students have specific interest in the subject and poor performing students complete fewer evaluations.^12^ As non-response rates increase, the likelihood that non-respondents’ opinions differ from respondents’ opinions increases.^9^ Therefore, low response rates are more likely to result in bias.

Some previous work has been done on ways to increase response rates to online evaluations by using a variety of methods. These methods include teachers making a concerted effort to promote the online evaluation, faculty providing students with information on the use of their feedback and entering student participants in a drawing for a cash prize.^13^ Small up-front gifts and conditional incentives have also been shown to increase response rates.^14^ In a review article, Nulty offered a set of twelve best practices for increasing response rates to online surveys and suggests that these method are additive (table 4). While some techniques demonstrate an increase in evaluation response, they are often dependent on the enthusiasm of the faculty and response rates decline as time passes and enthusiasm wanes.^15^

While not supported by the literature, there is concern that making evaluations mandatory may affect evaluation quality. In addition, most methods that have been shown to increase response rates require input or effort by the preceptor or by an administrator. The benefit of the tit-for-tat method is it uses an automated system completely independent of additional input or effort and it accomplishes an increase in response rates through incentives. Therefore, this study supports the hypotheses that a tit-for-tat incentive strategy does increase the total number of preceptor evaluations submitted by medical students without making evaluation submission mandatory.

## LIMITATIONS

Unfortunately, the evaluation system used did not track demographic data. The demographics of each study year are approximately similar to the graduating class of each academic year, but due to the presence of away rotators, the demographics cannot be calculated accurately. Differences in demographics could exist between the students in each academic year and possibly skew the results. Further, we also have no data on the number of student assessments completed by preceptors. It is unclear if there were fluctuations in the number of student assessments between academic years or what affect those fluctuations would have if they exist. During this time there were minor increases in the number of faculty and residents, it is unclear what effect this may have had. In addition, our data also shows that that average evaluation score of the emergency medicine rotation steadily increased over the course of the data collection period. It is possible that the increased popularity could have contributed to the response rates of the evaluation. As previously discussed, students are more likely to complete evaluations in subjects of personal interest. It should also be noted that this study demonstrated that the increase occurred after the intervention and concluding a causal relationships from this before and after study has limitations. Finally, the authors have no data on the quality of the evaluations. It is possible that the additional submitted evaluations differ in usefulness of comments and thus have affect the utility of the intervention.

## CONCLUSION

A significantly increased in the number of evaluations submitted by students rotating through their emergency medicine clerkship was observed following the implementation of the tit-for-tat method. This served as an effective tool to increase the number of evaluations completed by students and the proportion of students that completed evaluations. Further work should be done to identify any affect of the tit-for-tat method on the quality of evaluations and better understand additional methods of increasing evaluation response rates and assess if these methods are summative.

## Figures and Tables

**Table 1:** Breakdown of evaluations by academic year.

	Total # of student rotators	# of student evaluators	#of faculty evaluated	#of residents evaluated	Total # of evaluation
Academic year					
2012–13	153	99	100	48	489
2013–14	153	102	101	42	612
2014–15	171	161	123	59	1435
2015–16	163	146	125	57	1301
Control cohort					
2012–14	306	201	201	90	1101
Tit-for-tat cohort					
2014–16	334	307	248	116	2736
2012–16	640	508	449	206	3837

**Table 2:** Evaluations completed per student.

	# of student rotators	Mean # of evaluations per student	Standard deviation
Faculty control	306	2.11	2.593
Faculty for tit-for-tat	334	4.85	2.621
Resident control	306	1.49	1.769
Resident tit-for-tat	334	3.34	2.244
All preceptor control	306	3.60	3.959
All preceptor tit-for-tat	334	8.19	3.791

**Table 3:** Evaluations received per preceptor.

	# of student rotators	Mean # of evaluations per student	Standard deviation
Faculty control	201	3.21	2.16
Faculty for tit-for-tat	248	6.53	4.924
Resident control	91	5.01	3.421
Resident tit-for-tat	116	9.63	6.345
All preceptor control	292	3.77	2.743
All preceptor tit-for-tat	364	7.52	5.599

**Table 4:** Best practices for increasing response rates to online surveys.

Push the survey Provide frequent reminders Involve faculty in frequent emphasis of importance Persuade respondents that their responses will be used Provide rewards Help students understand how to give constructive criticism Create surveys that seek constructive criticism Extend the duration of a survey’s availability Involve students in the choice of optional questions Assure students of the anonymity of their responses Familiarize students with online evaluation environment Keep questionnaires brief
